# Does Hypoxia Decrease the Metabolic Rate?

**DOI:** 10.3389/fendo.2018.00668

**Published:** 2018-11-13

**Authors:** Chenjuan Gu, Jonathan C. Jun

**Affiliations:** Division of Pulmonary and Critical Care, Department of Medicine, Johns Hopkins University, Baltimore, MD, United States

**Keywords:** hypoxia, metabolism, oxygen, metabolic rate, temperature

## Abstract

In some organisms and cells, oxygen availability influences oxygen consumption. In this review, we examine this phenomenon of hypoxic hypometabolism (HH), discussing its features, mechanisms, and implications. Small mammals and other vertebrate species exhibit “oxyconformism,” a downregulation of metabolic rate and body temperature during hypoxia which is sensed by the central nervous system. Smaller body mass and cooler ambient temperature contribute to a high metabolic rate in mammals. It is this hypermetabolic state that is suppressed by hypoxia leading to HH. Larger mammals including humans do not exhibit HH. Tissues and cells also exhibit reductions in respiration during hypoxia *in vitro*, even at oxygen levels ample for mitochondrial oxidative phosphorylation. The mechanisms of cellular HH involve intracellular oxygen sensors including hypoxia-inducible factors, AMP-activated protein kinase (AMPK), and mitochondrial reactive oxygen species (ROS) which downregulate mitochondrial activity and ATP utilization. HH has a profound impact on cardiovascular, respiratory, and metabolic physiology in rodents. Therefore, caution should be exercised when extrapolating the results of rodent hypoxia studies to human physiology.

## Background

Hypoxia is defined as reduced oxygen (O_2_) in the environment or in an organism (1). Arterial hypoxia is detected by O_2_ sensitive cells primarily located in the carotid body. Activation of the carotid bodies stimulates hyperventilation and activates the sympathetic nervous system. Peripheral tissues also mount local responses to hypoxia. For example, skeletal muscle vasculature dilates to permit greater blood flow (2). Reduced oxygen in the kidney and liver tissues upregulates the expression of erythropoietin, leading to increased hemoglobin. Angiogenesis is stimulated by growth factors such as vascular endothelial growth factor 1. Thus, hypoxia activates several systems that increase O_2_ delivery.

Another defense against hypoxia is a downregulation of metabolic rate/O_2_ demand. Throughout the animal kingdom, both vertebrates and invertebrates can dramatically reduce metabolic rate and body temperature (T_b_) in response to cold or reduced O_2_ levels. Hypoxia reduces T_b_ in both endothermic (e.g., mammals) and ectothermic (e.g., reptiles) vertebrates (3). In hibernating mammals, metabolism can reversibly fall to 2% of basal metabolic rate (BMR) (4, 5). The drop in metabolic rate during hypoxia was defined as “Hypoxic hypometabolism” by Mortola et al. in the early 1990s (6). This hypometabolic state conserves oxygen stores (7) and protects against ischemic injury after cardiac arrest (8). Freshwater turtles can survive for months with minimal O_2_ during winter hibernation (9). In this review, we will examine features, mechanisms, and implications of hypoxic hypometabolism (HH) and hypoxia-induced hypothermia.

## Hypoxic hypometabolism

“Oxyregulators” are organisms that maintain metabolic rate regardless of O_2_ availability, while “oxyconformers” decrease energy expenditure in the face of lower O_2_ availability (6)_._ Most mammals including humans are oxyregulators: as O_2_ supply decreases (e.g., ischemia, exercise) anaerobic pathways supply ATP to compensate for the O_2_ debt. Other vertebrates, exemplified by the crucian carp, and common frog primarily use HH to reduce ATP demand (10). Small mammals (newborn humans, kittens, rats, adult guinea pigs) are oxyconformers, lowering their oxygen consumption ($\dot{\text{V}}$O_2_), and T_b_, even in relatively mild hypoxia (11). Studies in the 1950's identified two factors that influenced the magnitude of HH in mammals: ambient temperature and body mass (12). Hypoxia inhibits shivering and non-shivering thermogenesis, which are costly energetic processes. The thermoneutral zone (TNZ) is the range of ambient temperatures where BMR is determined, since thermogenic energy expenditure is at a minimum (13). Seminal studies by Hill (14) elegantly showed that $\dot{\text{V}}$O_2_ and T_b_ decreased in newborn kittens exposed to hypoxia (10% O_2_) at an ambient temperature of 28°C (below TNZ), but not in the TNZ (34°C). The effect has now been termed regulated hypothermia, or hypoxia-induced anapyrexia (15), and involves selection of a cooler environment (16), increased dissipation of heat (17), suppression of shivering (18, 19), and inactivation of brown fat thermogenesis (20).

The relatively large surface area to body mass of small mammals causes substantial heat dissipation, requiring a high BMR that rises rapidly for each degree below TNZ (21, 22). Frappell et al. compared several newborn mammals and found that mammals weighing >2 kg exhibited minimal HH or hypoxic hypothermia (23). Larger mammals have a lower weight-adjusted BMR, reduced thermosensitivity, a lower TNZ, and a blunted rise of $\dot{\text{V}}$O_2_ per degree below TNZ (24). The TNZ of a human lies in 18–22°C (clothed) or 25–30°C (unclothed) range. The TNZ of mice is ~30–34°C. Thus, “room temperature” (22°C) approximates the TNZ for clothed humans but is far below TNZ for mice; the metabolic rate of a mice housed at 22°C will be 50% above its BMR (13).

## Mechanisms of hypoxic hypometabolism

Features of HH and hypoxia-induced hypothermia have been characterized in detail, but underlying mechanisms are not fully understood. It is clear that HH is not caused by anaerobic metabolism or “oxygen debt” (23), which suggests that HH is a regulated process. Tamaki and Nakayama showed that preoptic hypothalamic neurons became less temperature sensitive in anesthetized rats when exposed to 10% O_2_ (25). Tattersall and Milsom showed that the threshold for hypothalamic activation to central cooling decreased from 38°C in normoxia to 28~31°C at 7% O_2_ by delivering a cold stimulus to the brains of ground squirrels using implanted thermodes (12). Less certain is how hypoxia is sensed by the hypothalamus leading to HH. Matsuoka et al. (26) reported that anemic hypoxia (normal PaO_2_) reduced $\dot{\text{V}}$O_2_ in rats, indicating that HH does not require activation of the carotid body. This provides circumstantial evidence that O_2_-sensitive neural networks in the brainstem, already known to regulate respiratory and sympathetic activity (27) may relay information to the preoptic hypothalamus. How O_2_ is sensed within cells involves several regulatory proteins, ion channels, and mitochondrial reactive oxygen species (ROS) (28). Adenosine may increase during hypoxia and signal thermoregulatory changes at the preoptic hypothalamus. Barros et al. found that an adenosine A1 receptor antagonist attenuated HH in rats (29, 30). Hypoxia-induced anapyrexia may also involve neurotransmitters such as hydrogen sulfide (31), endogenous opioids (32), and nitric oxide (33). Effectors of heat generation may also be affected by local O_2_ tension. Hypoxia causes the stabilization of hypoxia-inducible factor-1 (HIF-1), which controls the transcription of many genes. HIF-1 may suppress O_2_ consumption and heat production from brown adipose tissue (20). It is estimated that resting $\dot{\text{V}}$O_2_ decreases by 11% per degree fall in T_b_ due to slower enzymatic reaction rates (the “Q_10_” effect) (3). However, the fact that $\dot{\text{V}}$O_2_ decreases before T_b_, and falls to a greater extent than predicted by Q_10_ effects, argues against hypothermia *per se* causing HH. Regardless of the pathways involved, HH and lower T_b_ act synergistically to increase survival (11). In summary, animals that exhibit HH conform their metabolic demands to reduced O_2_ availability sensed in brain.

## Hypoxic hypometabolism at the cell level

The above discussion focused on the decrease in whole body $\dot{\text{V}}$O_2_ and T_b_ in response to hypoxia. Oxygen conformism also occurs at the cellular level (10). Below a critical anoxic threshold, cell death occurs if O_2_ availability fails to meet ATP demands of Na-K-ATPases and voltage-gated Ca^2+^ channels. Cells from different species and organs exhibit varying levels of anoxia tolerance. Organisms that exhibit significant hypoxia tolerance are comprised of cells capable of suppressing activity of ion-motive ATPases, a protein pump which allows ions to move against the electrochemical potential gradient across biological membranes at the expense of ATP hydrolysis. This phenomenon is termed “channel arrest” (34). Turtle and frog tissues (liver, heart, brain) can reversibly reduce respiratory rates by 75% within 30 min of exposure to anoxia (10). Similarly, hypoxia can induce complete and reversible arrest of mitochondrial respiration and ATP synthesis in liver cells of diving seals (35). Mechanisms of channel arrest in anoxia tolerant cells is not known, but may involve adenosine accumulation as a signaling molecule. By contrast, cells from oxyregulators do not exhibit decreases in ATP demand for maintaining ion gradients (10).

Cellular respiration decreases even at moderately reduced O_2_ levels (1–3%), well above the threshold (<0.3% O_2_) for arresting O_2_-dependent ATP generation at mitochondrial complex IV (36). For example, isolated rat hepatocytes exhibited a reversible fall in $\dot{\text{V}}$O_2_ after several hours of exposure to ~10% O_2_ (37); chick cardiac myocytes also exhibited HH and decreased contractility with evidence of reduced mitochondrial complex IV activity (38). Under normoxic conditions, cellular $\dot{\text{V}}$O_2_ in cells is determined by factors including rates of ATP synthesis, transport and utilization (50%), NADH supply generated from pyruvate flux and the tricarboxylic acid (TCA) cycle (15–30%), proton leak (0–15%), and the electron transport chain (ETC) (39). These processes are not affected by brief hypoxia, but within a few hours, carbon flux through the TCA, and electron flux through the ETC both decrease (36). Cellular HH is mediated in part through stabilization of HIF-1α. HIF-1 shifts metabolism toward glycolysis via upregulating numerous glycolytic genes (40, 41), a phenomenon called the Pasteur effect (42). HIF-1 actively suppresses the TCA cycle by trans-activating the gene encoding pyruvate dehydrogenase kinase 1 (PDK1), which inactivates pyruvate dehydrogenase (PDH). PDH is responsible for the conversion of pyruvate to acetyl-CoA. The net result is a shunting of pyruvate away from the TCA cycle and toward glycolysis, as well as a fall in mitochondrial $\dot{\text{V}}$O_2_ and an increase in intracellular O_2_ tension (43, 44).

Reduced electron flux through the ETC during sustained hypoxia occurs via several mechanisms, some of which are dependent upon HIF-1. First, HIF-1 targets inducible nitric oxide synthase (iNOS), and nitric oxide in turn suppresses mitochondrial complex IV activity. Second, HIF-1 stimulates micro-RNA 210, which inhibits the function of several mitochondrial membrane complexes. Third, HIF-1 induces a switch of subunits expressed in complex IV, which increases its efficiency (45). Another major mechanism of HH involves inhibition of ATP utilization. Hypoxia inhibits plasma membrane Na-K-ATPase activity, which may account for up to 70% of mammalian cellular $\dot{\text{V}}$O_2_ (46). Hypoxia (1.5% O_2_) was shown to cause ubiquitin degradation of the Na-K-ATPase alpha subunit (47). In addition, hypoxia inhibits cellular mRNA translation. The reduction of Na-K-ATPase activity and protein translation are both mediated by an O_2_ sensor, AMP-activated protein kinase (AMPK) which is activated by mitochondrial ROS (36). Mechanisms of HH at the cellular level are complex and remain under active investigation.

## Do humans experience hypoxic hypometabolism?

Humans (other than newborns) would be classified as oxyregulators, and do not exhibit HH. In fact, the cardiovascular stress of hypoxia is often accompanied by changes such as hyperventilation that increase O_2_ delivery and increase $\dot{\text{V}}$O_2_. Exposure to high altitude (hypobaric hypoxia) is accompanied by weight loss, with increased energy expenditure being one of the mechanisms (48). For example, the metabolic rate of male sea level natives increased 27% on day 2 after ascent to 4,300 m, and remained 17% higher than baseline on day 10 (49). High altitude exposure also increases rates of glucose turnover in the body, at rest and during exercise (50). BMR of workers residing in the Andes (~4,500 m) for >4 months showed values comparable to standard BMR measurements at sea level, and higher than sea level values when normalized to lean body mass (51). This finding is in line with older studies of acute high altitude exposure showing a rise in $\dot{\text{V}}$O_2_ (52). Interestingly, six scientific expeditioners to the Himalayas (5,800 m) showed a 10% increase, while their 3 Sherpa guides (chronic dwellers at 1,800 m) exhibited a 21% increase in $\dot{\text{V}}$O_2_ compared to sea level standards (53). Normobaric hypoxia (breathing 10% O_2_ for 40 min) resulted in a 15.5% increase in cerebral blood flow and 8.5% increase in cerebral metabolic rate in healthy subjects, as measured by magnetic resonance imaging (54).

Hypoxia lowers peak $\dot{\text{V}}$O_2_ and causes an earlier shift to anaerobic metabolism during intensive exercise (55–57). However, this lowering of $\dot{\text{V}}$O_2max_ should not be equated with HH. $\dot{\text{V}}$O_2_ continues to rise at work rates above anaerobic threshold. Thus, humans and larger mammals cope with hypoxia by “defending” ATP production rather than conforming to a lower $\dot{\text{V}}$O_2_. It is possible that long-term hypoxic adaptation can induce changes in metabolism of certain tissues. For example, Hochachka et al. (58) examined brain regional glucose metabolic rates in Quechua natives indigenous to the Andes (3,700–4,900m), with positron emission tomographic imaging. These high-altitude dwellers demonstrated lower glucose metabolic rates than that of lowlanders. However, there is no evidence that acute or chronic hypoxia reduces overall $\dot{\text{V}}$O_2_ in humans.

## Do humans experience hypoxia-induced anapyrexia?

It is unclear whether hypoxia significantly alters thermoregulation in humans. DiPasquale et al. studied subjects breathing 21, 14, or 12% O_2_ for 30 min at thermoneutrality. They showed that hypoxia modestly decreased rectal temperature, with each 1% decrease in SpO_2_ decreasing the temperature by 0.15°C (59). However, Seo et al. questioned possible carryover effects of the brief interventions of this study which were performed in a single session per subject. Their group studied thermal responses to similar degrees of normobaric hypoxia, distributing exposures across days, and did not find a reduction in rectal temperature, metabolic heart production, or heat loss (60). Other studies used cold exposure to elicit changes in thermoregulation with hypoxia. Eight subjects immersed in 28°C water were exposed to eucapnic hypoxia (12% O_2_) which lowered core temperature threshold for vasoconstriction and shivering by 0.14 and 0.19°C, respectively, while increasing core cooling rate (61). Robinson and Haymes exposed subjects to normoxia or hypoxia (12% O_2_), at an ambient temperature of 25 or 8°C. Under cold conditions, hypoxia modestly lowered $\dot{\text{V}}$O_2_ and rectal temperature. During cold exercise, hypoxia accelerated heat loss (62). Another study examined the acute effects of normobaric hypoxia on hand temperature responses during and after a 30-min local cold-water immersion test. Although hypoxia did not aggravate the cold-induced drop in hand temperature, hypoxia impaired rewarming (63).

Some studies examined thermoregulation at high altitude, an environment that often combines hypobaric hypoxia with cold temperature. Savourey et al. (64) studied 11 lowlander subjects after 2 weeks of high altitude residence in the Andes (4,150 ~ 6,885 m). Metabolic heat production in response to a cold air test (2 h of 1°C exposure) was modestly diminished and heat debt increased, whereas upper-extremity skin temperature was reduced by ~1.45°C in a local coldwater test (5 min of 5°C exposure) after 2 weeks at high altitude. In study that controlled for ambient temperature, five men were exposed to acute intermittent hypoxia (AIH) in a chamber (8 h daily for 4 d, 6 h on the last day, 4,500–6,000 m) at 24°C. Under these conditions, cold challenge testing demonstrated that AIH caused lower skin temperature, without significant change in rectal temperature. Interestingly, metabolic heat production increased by 7% and heat debt and convective heat loss decreased. Time to onset for continuous shivering also decreased (65). O'Brien et al. (66) performed finger cold water immersion tests in healthy males in a thermoneutral hypobaric chamber at simulated sea level, 3,000 and 4,675 m. No effect of hypobaric hypoxia on the finger temperature response was observed. In summary, hypoxia may impair thermoregulation in adult humans, but effects are small and may require superimposed cold exposure to become evident.

## Implications of hypoxic hypometabolism

### Animal physiology

HH influences cardiovascular and respiratory physiology of small mammals. Mice housed at typical lab temperature (22°C) exhibit high sympathetic activity, low cardiac vagal tone, and a higher resting heart rate compared to mice housed at TNZ (67). In a rodent study using three different species (rats, ground squirrel, and hamster), hypoxia resulted in cardiac acceleration in all species in a warm environment (35°C), while decreasing heart rate at an ambient temperature of 10°C (68). Similarly, the magnitude of the hypoxic ventilatory response (HVR) is modified by HH (69). When rats of different sizes were exposed to 10% O_2_ at an ambient temperature of ~24°C, 400 g rats had much stronger HVR than 50 g rats associated with a minimal degree of HH in the larger animals (70). More directly it was shown that inhaled hydrogen sulfide induced HH in mice, and mediated a reduction in HVR (71). Substrate metabolism under hypoxic conditions is also highly influenced by ambient temperature. To see if acute hypoxia increases plasma triglycerides (TG), we exposed postprandial mice housed at 22°C to 6 h of graded hypoxia. Hypoxia dose-dependently increased TG [as seen in a previous rat studies (72, 73)] contained within large, low-density lipoproteins while decreasing TG clearance, and decreasing fatty acid uptake in brown adipose tissue (74). When mice were exposed to 10% O_2_ at thermoneutrality (30°C) hypoxia had no effect on TG levels, clearance rate, or brown adipose tissue lipid uptake. Moreover, thermoneutral hypoxia increased cardiac lipid uptake and plasma HDL cholesterol (75). Baum et al. found that hypoxia inhibited lipolysis in puppies exposed to cold (76). However, hypoxia stimulated lipolysis in mice under thermoneutral conditions (75, 77) with more variable responses below TNZ (74).

### Translational research

Small mammal studies performed below TNZ would indicate that hypoxia lowers $\dot{\text{V}}$O_2_ (14), reduces heart rate (68), minimally increases ventilation (70), causes an atherogenic lipid profile (72, 73), and inhibits lipolysis (76). However, many of these changes are manifestations of cold-elicited HH. Hypoxia in rodents at TNZ better approximates the human response, characterized by a preserved $\dot{\text{V}}$O_2_, robust HVR and heart rate acceleration, no change (78) or reduced TG (75), increased HDL cholesterol (79), and a stimulation of adipose tissue lipolysis (78, 80, 81). Therefore, ambient temperature is a critical variable in translational hypoxia studies. To “humanize” small mammal hypoxia research, HH can be minimized by housing animals at TNZ.

### Clinical research

Understanding HH may have clinical applications. Downregulation of metabolism is evident in myocardium during ischemia (82). Pre-conditioning tissues to hypoxia may mitigate ischemia-reperfusion injury (83). Cancer cells invoke HH to promote survival in hypoxic tumors (84, 85). HH may be an adaptive strategy for neonatal humans at risk for sudden infant death (86). Therefore, pathways of HH may be leveraged for human disease.

## Conclusions

In oxygen “conformers” hypoxia can reduce metabolic rate, at the whole body and cellular level. Factors that determine the extent of HH include the degree of hypoxia, ambient temperature, body mass, and species or cell type. Knowledge of these factors is critical for the design and interpretation of hypoxia studies. The ability to manipulate HH may also have significant therapeutic implications.

## Author contributions

CG and JJ wrote the manuscript and had final approval of the submitted version.

### Conflict of interest statement

The authors declare that the research was conducted in the absence of any commercial or financial relationships that could be construed as a potential conflict of interest.
